# Nonlinear Robust Control by a Modulating-Function-Based Backstepping Super-Twisting Controller for a Quadruple Tank System

**DOI:** 10.3390/s23115222

**Published:** 2023-05-31

**Authors:** Italo Aranda-Cetraro, Gustavo Pérez-Zúñiga, Raul Rivas-Pérez, Javier Sotomayor-Moriano

**Affiliations:** 1 Departamento de Ingeniería, Pontificia Universidad Católica del Perú (PUCP), Avenida Universitaria 1801, San Miguel, Lima 15088, Peru; 2 Departamento de Automática y Computación, Universidad Tecnológica de la Habana José Antonio Echeverría (CUJAE), La Habana 19390, Cuba

**Keywords:** variable structure systems, modulating functions, MIMO systems, backstepping control, nonlinear control, quadruple tank system

## Abstract

In this paper, a robust nonlinear approach for control of liquid levels in a quadruple tank system (QTS) is developed based on the design of an integrator backstepping super-twisting controller, which implements a multivariable sliding surface, where the error trajectories converge to the origin at any operating point of the system. Since the backstepping algorithm is dependent on the derivatives of the state variables, and it is sensitive to measurement noise, integral transformations of the backstepping virtual controls are performed via the modulating functions technique, rendering the algorithm derivative-free and immune to noise. The simulations based on the dynamics of the QTS located at the Advanced Control Systems Laboratory of the Pontificia Universidad Católica del Perú (PUCP) showed a good performance of the designed controller and therefore the robustness of the proposed approach.

## 1. Introduction

Currently, there are a considerable number of industrial multivariable processes with complex nonlinear dynamic behavior [1,2,3]. Therefore, multivariable control strategies are highly important and have received significant attention from the international scientific community; see, for example, [4,5,6,7,8,9].

The quadruple tank system (QTS) has been widely used in academia to design multi-input, multi-output (MIMO) control schemes for liquid level regulation in the presence of complex nonlinear dynamics [10]. Classical control approaches to this problem involve linearizing the model about an operating point (OP) and using decouplers to eliminate or reduce the interaction sensitivity between inputs and outputs, in order to apply decentralized proportional-integral (PI) control [11]. In [12], an actuator fault-tolerant decentralized PI-controller based on the design of simplified decouplers was proposed, allowing feed-forward control, as if it were two independent single input single output (SISO) systems [13]. The fault-tolerant part of the controller had the ability to additively compensate for the magnitude of the fault. Yet, one of the downsides of this approach was that the designed decouplers were not always physically feasible and were prone to modeling errors [12].

In [14], the performance of a decentralized PI controller was compared to robust control strategies such as a multivariable internal model control (IMC) and an H-infinity control (H∞), concluding that these control strategies yielded better performance than the decentralized PI controller. In [15], a reconfigurable model predictive control (MPC) approach was followed, where the feasibility to compensate for the control signal was shown when an actuator (pump) failure was detected by switching its control signal to another actuator and switching to the output of an observer when a sensor fault was detected. In general, all these methods limited their overall performance to an operating point, since they worked with a linearized model of the system.

However, other authors proposed working directly with the variable structure control (VSC) of nonlinear multivariable systems. Variable structure control systems (VSCS) theory, based on the phase-plane method of the oscillatory theory, was proposed by Alexander Andronov in the 1940s [16]. This control theory was formally presented by Stanislav Emelyanov in the 1960s and developed by Vadim Utkin [17] and other authors afterwards. A VSCS is composed of continuous subsystems and a commutation law between these subsystems, originating discontinuous or bang–bang control efforts to stabilize or regulate the response of the processes.

Sliding mode control (SMC) is a special type of VSCS, since it introduces an error variable named a “sliding variable” to steer the trajectory of the system to a sliding manifold and maintain motion on the manifold by means of discontinuous control, regardless of disturbances or uncertainties to the process [18]. Nonetheless, due to its inherent robustness, the SMC introduces chattering or high-frequency oscillations to the control, which is undesirable for actuators. Various chattering-suppression methods have been proposed [19], with the equivalent control method [17] and higher-order sliding modes (HOSM) [20] as the most representative. Moreover, in [21] it was proved that the first-degree SMC and HOSM were sensitive to non-Gaussian measurement noise, making it necessary to test and implement special HOSM differentiator algorithms and filters to mitigate the influence of the nonlinear noise distributions in the sliding variable for real applications.

Concerning the use the use of the SMC and HOSM algorithms for liquid level regulation of the QTS, in [22], an SMC technique through feedback linearization was proposed, yielding better performance than the conventional PI controller. In [23], a second-order sliding mode (SOSM) controller based on the twisting algorithm (TA) was designed to regulate the liquid levels while considerably reducing the chattering level of the control effort. Even though the SOSM and HOSM methods proved to be effective in reducing the chattering level, they did not completely suppress it in some cases.

In [24], the active disturbance rejection control (ADRC) was introduced as an alternate approach to PID control, based on the design of a tracking differentiator, which provided the error signal, the derivative of the error signal, and a sliding mode controller (SMC). Recently, improvements were made in [25] with regard to the ADRC based on the work provided in [24] with promising results, although the performance of the tracking differentiator was not addressed in the presence of measurement noise.

The main contribution of this paper consists in the proposal of a robust nonlinear approach for control of liquid levels in a quadruple tank system (QTS) based on the combination of a backstepping controller and a super-twisting controller (BSSTC), implementing a multivariable sliding surface, where the error trajectories converge to the origin at any operating point of the system. Because the backstepping algorithm is dependent on the derivatives of the state variables and is sensitive to measurement noise, integral transformations of the backstepping virtual controls are carried out using modulating functions technique, making the algorithm derivative-free and immune to noise. All the modeling and control methodologies shown in this paper have been developed for a real laboratory QTS.

This paper proceeds as follows. In Section 2, a mathematical model of the process studied is obtained using modeling techniques. The theoretical background is explained in Section 3. The design of the MF-BSSTC controller is developed in Section 4. The discussions of the attained results are presented in Section 5. Lastly, a few conclusions are given in Section 6.

## 2. Quadruple Tank System Modeling

**Figure 1:**
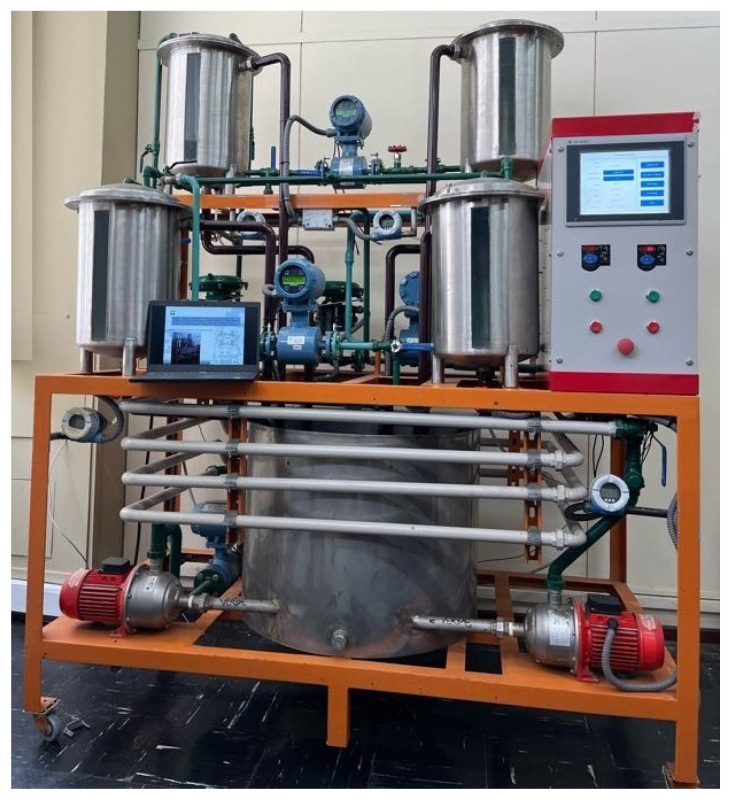
Quadruple tank system of the PUCP’s Advanced Control Systems Laboratory.

**Figure 2:**
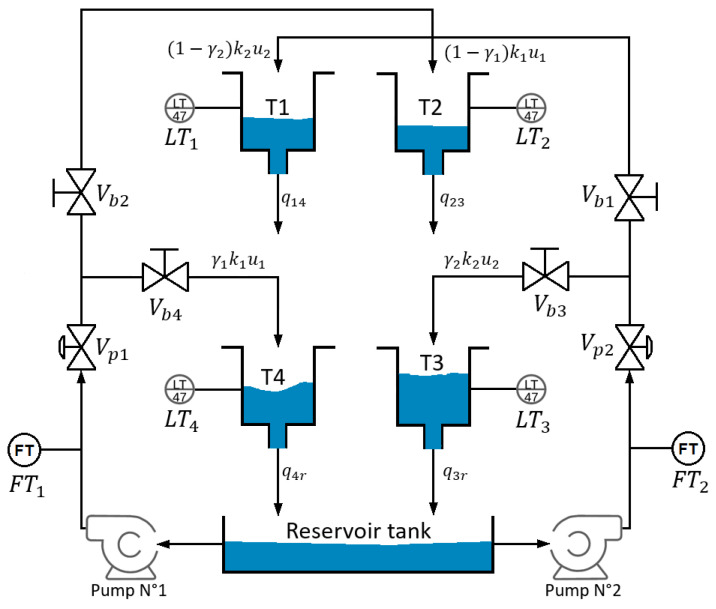
Quadruple tank system diagram.

The study presented in this paper is based on the QTS located at PUCP’s Advanced Control Systems Laboratory. This QTS consists of four coupled tanks, a reservoir tank, four ball valves, two proportional valves, and two pumps. Figure 1 shows a view of this QTS, and its schematic representation is exhibited in Figure 2.

### QTS Modeling under Disturbances

The control objective is to regulate the liquid levels of tanks n°3 and n°4. The inputs to the system are the flow rates $u_{1}$ and $u_{2}$ from pumps n°1 and n°2, respectively, measured by the flow transmitters $FT_{1}$ and $FT_{2}$. The measured outputs are the liquid levels $h_{1}$, $h_{2}$, $h_{3}$, and $h_{4}$ of tanks n°1, n°2, n°3, and n°4, respectively, which are proportional to the voltages generated by the level transmitters $LT_{1}$, $LT_{2}$, $LT_{3}$, and $LT_{4}$. The system has two proportional valves $V_{p1}$ and $V_{p2}$ with aperture percentages $k_{1}$ and $k_{2}$ ∈[0,1], respectively, two ball valves $V_{b2}$ and $V_{b4}$, which generate flow towards tanks n°2 and n°4 with a split constant $\gamma_{1}\in \left[0,1\right]$, and two ball valves $V_{b1}$ and $V_{b3}$ which generate flow towards tanks n°1 and n°3 with a split constant $\gamma_{2}\in \left[0,1\right]$. Since the output flow rates of pump n°1 split between tank n°2 and tank n°4 and the output flow of pump n°2 between tank n°1 and tank n°3, the position of valves $\gamma_{1}$ and $\gamma_{2}$ controls the split ratio. For instance, if $0<\gamma_{1}+\gamma_{2}<1$ holds, one transmission zero out of the two transmission zeros of the system locates at the right half plane (RHP) of the root locus, generating nonminimum phase dynamics. On the other hand, if $1<\gamma_{1}+\gamma_{2}<2$ holds, both transmission zeros locate at the left half plane (LHP) of the locus, rendering the QTS with minimum phase dynamics.

In [14], mass balances and Bernoulli’s law yield the following multivariable QTS model with disturbances:

(1) $$\begin{array}{cc} & \;{\dot{h}}_{1}=-\frac{a_{1}\sqrt{2gh_{1}}}{A_{1}}+\frac{\left(1-\gamma_{2}\right)k_{2}u_{2}}{A_{1}}+\xi_{1},\\ & \;{\dot{h}}_{2}=-\frac{a_{2}\sqrt{2gh_{2}}}{A_{2}}+\frac{\left(1-\gamma_{1}\right)k_{1}u_{1}}{A_{2}}+\xi_{2},\\ & \;{\dot{h}}_{3}=-\frac{a_{3}\sqrt{2gh_{3}}}{A_{3}}+\frac{a_{2}\sqrt{2gh_{2}}}{A_{3}}+\frac{\gamma_{2}k_{2}u_{2}}{A_{3}}+\xi_{3},\\ & \;{\dot{h}}_{4}=-\frac{a_{4}\sqrt{2gh_{4}}}{A_{4}}+\frac{a_{1}\sqrt{2gh_{1}}}{A_{4}}+\frac{\gamma_{1}k_{1}u_{1}}{A_{4}}+\xi_{4},\\ & \;y={\left[\begin{array}{cccc} h_{1} & h_{2} & h_{3} & h_{4} \end{array}\right]}^{\prime }+v, \end{array}$$

where $x={\left[\begin{array}{cccc} h_{1} & h_{2} & h_{3} & h_{4} \end{array}\right]}^{\prime }$ represents the state variables vector, $h_{i}$∀i=1,2,3,4 is the tank *i* liquid level in cm, *y* is the measurement vector with *v* as additive noise, $u={\left[\begin{array}{cc} u_{1} & u_{2} \end{array}\right]}^{\prime }$ is the control vector, $A_{i}$ is the cross section of tank *i* in cm${}^{2}$, $a_{i}$ is the cross section of the outlet hole of the tank *i* in cm${}^{2}$, $d_{i}$ is the diameter of the tank *i* in cm, ${h_{i}}_{max}$ is the maximum liquid level of tank *i*, and *g* is the acceleration of gravity in cm/s${}^{2}$. Moreover, $\xi_{1}=\frac{\left(1-\gamma_{2}\right)f_{p2}}{A_{1}}$, $\xi_{2}=\frac{\left(1-\gamma_{1}\right)f_{p1}}{A_{2}}$, $\xi_{3}=\frac{\gamma_{2}f_{p2}}{A_{3}}$, and $\xi_{4}=\frac{\gamma_{1}f_{p1}}{A_{4}}$ are disturbances to the system generated by the flow losses $f_{p1}$ and $f_{p2}$ of pump n°1 and pump n°2, respectively, which are modeled as step functions, such that $\dot{\xi_{i}}=0$. In Table 1, the parameter values of the four-tank coupled system are presented.

## 3. Theoretical Background

Sliding mode control (SMC) has the ability to reject bounded matched uncertainties at the cost of introducing chattering to the control input. This could be detrimental to the performance if the mechanical systems are controlled, as is the case with QTS. In fact, one of the many methodologies available to suppress chattering is the design of super-twisting algorithm (STA) based controllers [20], which has been extensively used in recent years for closed-loop control of the QTS.

A super-twisting controller (STC) is a type of SOSM applicable to a system, where the control appears in the first derivative of the sliding variable [26], which has the ability to compensate for disturbances or uncertainties with only the knowledge of the measured output or sliding variable σ, while suppressing or attenuating chattering. For instance, the STC could be applied to the QTS if a multivariable STC scheme was designed, considering that there should exist as many sliding surfaces as independent controls [27], and some “hierarchy of controls” [17] should be established.

**Definition 1**  (Multivariable Sliding Surface [27])**.** *Let the general state-space representation of a system with multiple controls be*

(2) $$\begin{array}{cc} & \;\dot{x}=f\left(x\right)+g\left(x\right)u,\\ & \;y=h(x), \end{array}$$

*where $x\in R^{n}$ is the state variable vector of dimension n, $u\in R^{m}$ is the control vector of dimension m, and $y\in R^{n}$ is the output vector of dimension n, where n and m represent the number of controlled systems and the number of sliding surface coordinate functions defined as system outputs, respectively. Then, the multivariable sliding surface is represented by the simultaneous satisfaction of m smooth algebraic state restrictions, summarized in the equation σ(x)=0, which represents the intersection manifold,*

(3) $$S=\left\{x\in R^{n}|\;\sigma_{i}\left(x\right)=0,i=1,2,..,m\right\}=\overset{m}{\underset{i=1}{⋂}}S_{i}.$$

*For instance, the following vector of sliding surfaces was chosen for the QTS,*

(4) $$\sigma \left(x\right)={\left[\begin{array}{cc} \sigma_{1}\left(x\right) & \;\sigma_{2}\left(x\right) \end{array}\right]}^{T}={\left[\begin{array}{cc} c_{1}e_{4} & c_{2}e_{3} \end{array}\right]}^{T},$$

*where $e_{i}=h_{i}-{h_{i}}^{*}$ are the errors between the actual liquid level $h_{i}$ and the desired level ${h_{i}}^{*}$, $c_{i}>0$ are constants, and ∀i=1,2,3,4. ${h_{i}}^{*}$ must be a continuously differentiable reference trajectory. If the reference trajectory is a step, then ${h_{i}}^{*}={{\dot{h}}_{i}}^{*}=\ldots =0$.*

Therefore, the simultaneous satisfaction of algebraic constraints $\sigma_{1}\left(x\right)$ and $\sigma_{2}\left(x\right)$, which geometrically represents the existence of a smooth intersection manifold *S*, ideally produces a desired closed-loop behavior of the system, where *x* ∈ *S* holds in finite time [26].

In order to satisfy the algebraic constraints shown above, a first-degree SMC and a BSSTC are designed.

### Synthesis of a First-Degree Sliding Mode Controller (SMC)

In this subsection a first-degree SMC is designed using the equivalent control method proposed in [17,27].

**Definition 2**  (Equivalent Control and Ideal Sliding Dynamics [27])**.** *Let the lie derivative $L_{g}\sigma \left(x\right)$ be locally invertible, where σ(x) is a vector of the sliding surfaces that satisfies σ(x)=0. Then,*

(5) $$\dot{\sigma }\left(x\right)=\frac{\partial \sigma (x)}{\partial x^{T}}\left(f\left(x\right)+g\left(x\right)u_{eq}\left(x\right)\right)=0,$$

*or*

(6) $$\dot{\sigma }\left(x\right)=L_{f}\sigma \left(x\right)+\left[L_{g}\sigma \left(x\right)\right]u_{eq}\left(x\right){|}_{\sigma =0}=0.$$

*Therefore, the equivalent control is expressed as*

(7) $$u_{eq}\left(x\right)=-{\left[L_{g}\sigma \left(x\right)\right]}^{-1}L_{f}\sigma \left(x\right){|}_{\sigma =0},$$

*with ideal sliding dynamics given by:*

(8) $$\dot{x}=f\left(x\right)-G\left(x\right){\left[L_{G}\sigma \left(x\right)\right]}^{-1}L_{f}\sigma \left(x\right).$$

The equivalent control is the smooth feedback control law, denoted by $u_{eq}\left(x\right)$, which ideally locally holds the state evolution in the smooth manifold ***S*** for any initial state of the system located locally in ***S*** [26]. However, the closed-loop controller obtained with the equivalent control method generates a phenomena called “chattering”, as mentioned above, which can potentially wear out actuators when applied. Thus, in this paper, two second-order super-twisting controllers (2-STC) are designed through the recursive nonlinear backstepping technique to generate smooth control actions to regulate the liquid level of the selected subsystems, in order to reach the smooth intersection manifold ***S*** at any point of operation without the need to linearize the system.

Taking the model (1) into the state-space representation introduced in (2), the matrices f(x) and g(x) are shown:

(9) $$f\left(x\right)=\left[\begin{array}{c} -\frac{a_{1}\sqrt{2gx_{1}}}{A_{1}}\\ -\frac{a_{2}\sqrt{2gx_{2}}}{A_{2}}\\ -\frac{a_{3}\sqrt{2gx_{3}}}{A_{3}}+\frac{a_{2}\sqrt{2gx_{2}}}{A_{3}}\\ -\frac{a_{4}\sqrt{2gx_{4}}}{A_{4}}+\frac{a_{1}\sqrt{2gx_{1}}}{A_{4}} \end{array}\right],$$

(10) $$g\left(x\right)=\left[\begin{array}{cc} 0 & \frac{\left(1-\gamma_{2}\right)k_{2}}{A_{1}}\\ \frac{\left(1-\gamma_{1}\right)k_{1}}{A_{2}} & 0\\ 0 & \frac{\gamma_{2}k_{2}}{A_{3}}\\ \frac{\gamma_{1}k_{1}}{A_{4}} & 0 \end{array}\right],$$

where $x_{i}=h_{i}\;\forall \;i=1,2,3,4$.

Let the sliding surfaces of the multivariable closed-loop control be $\sigma_{1}=x_{4}-{x_{4}}^{*}$ and $\sigma_{2}=x_{3}-{x_{3}}^{*}$. So, the lie derivatives of $\sigma_{1}$ and $\sigma_{2}$ along the direction of vector fields f(x) and g(x) are computed as follows:

(11) $$\begin{array}{cc} & \;L_{f}\sigma \left(x\right)=\left[\begin{array}{c} L_{f}\sigma_{1}\left(x\right)\\ L_{f}\sigma_{2}\left(x\right) \end{array}\right]=\left[\begin{array}{c} \frac{\partial \sigma_{1}\left(x\right)}{\partial x^{T}}f\left(x\right)\\ \frac{\partial \sigma_{2}\left(x\right)}{\partial x^{T}}f\left(x\right) \end{array}\right]=\left[\begin{array}{cccc} 0 & 0 & 0 & 1\\ 0 & 0 & 1 & 0 \end{array}\right]\left[\begin{array}{c} -\frac{a_{1}\sqrt{2gx_{1}}}{A_{1}}\\ -\frac{a_{2}\sqrt{2gx_{2}}}{A_{2}}\\ -\frac{a_{3}\sqrt{2gx_{3}}}{A_{3}}+\frac{a_{2}\sqrt{2gx_{2}}}{A_{3}}\\ -\frac{a_{4}\sqrt{2gx_{4}}}{A_{4}}+\frac{a_{1}\sqrt{2gx_{1}}}{A_{4}} \end{array}\right],\\ & \;L_{f}\sigma \left(x\right)=\left[\begin{array}{c} -\frac{a_{4}\sqrt{2gx_{4}}}{A_{4}}+\frac{a_{1}\sqrt{2gx_{1}}}{A_{4}}\\ -\frac{a_{3}\sqrt{2gx_{3}}}{A_{3}}+\frac{a_{2}\sqrt{2gx_{2}}}{A_{3}} \end{array}\right], \end{array}$$

(12) $$\begin{array}{cc} & \;L_{g}\sigma \left(x\right)=\left[\begin{array}{c} L_{g}\sigma_{1}\left(x\right)\\ L_{g}\sigma_{2}\left(x\right) \end{array}\right]=\left[\begin{array}{c} \frac{\partial \sigma_{1}\left(x^{T}\right)}{\partial x}g\left(x\right)\\ \frac{\partial \sigma_{2}\left(x^{T}\right)}{\partial x}g\left(x\right) \end{array}\right]=\left[\begin{array}{cccc} 0 & 0 & 0 & 1\\ 0 & 0 & 1 & 0 \end{array}\right]\left[\begin{array}{cc} 0 & \frac{\left(1-\gamma_{2}\right)k_{2}}{A_{1}}\\ \frac{\left(1-\gamma_{1}\right)k_{1}}{A_{2}} & 0\\ 0 & \frac{\gamma_{2}k_{2}}{A_{3}}\\ \frac{\gamma_{1}k_{1}}{A_{4}} & 0 \end{array}\right],\\ & \;L_{g}\sigma \left(x\right)=\left[\begin{array}{cc} \frac{\gamma_{1}k_{1}}{A_{4}} & 0\\ 0 & \frac{\gamma_{2}k_{2}}{A_{3}} \end{array}\right]. \end{array}$$

Then, replacing (11) and (12) in Equation (7), the equivalent control $u_{eq}$ to ensure σ(x)→0 in finite time is found as follows:

(13) $$u_{eq}\left(x\right)=\left[\begin{array}{c} \frac{a_{4}\sqrt{2gx_{4}}-a_{1}\sqrt{2gx_{1}}}{\gamma_{1}k_{1}}\\ \frac{a_{3}\sqrt{2gx_{3}}-a_{2}\sqrt{2gx_{2}}}{\gamma_{2}k_{2}} \end{array}\right],$$

with the following control law:

(14) $$u\left(x\right)=\left|\eta \right|\frac{1}{2}(\left[\begin{array}{c} 1\\ 1 \end{array}\right]-sign{\left({\sigma (x)}^{T}L_{g}\sigma \left(x\right)\right)}^{T},$$

where $\left|\eta \right|=1718\;{\mathrm{cm}}^{3}/s$ is the upper saturation threshold value for the flow rate, which equates to 60% of the maximum capacity of the pump. Furthermore, this control law u(x)=0 takes the lower saturation threshold value of 10% of the pump capacity when it is equal to 0.

**Proof.**  **Robust closed-loop stability:** Replacing Equation (13) in model (1), the following closed-loop dynamics are obtained:

(15) $$\begin{array}{cc} & \;{\dot{x}}_{1}=\frac{-\gamma_{2}a_{1}\sqrt{2gx_{1}}+\left(a_{3}-\gamma_{2}a_{3}\right)\sqrt{2gx_{3}}-\left(a_{2}-\gamma_{2}a_{2}\right)\sqrt{2gx_{2}}}{A_{1}\gamma_{2}},\\ & \;{\dot{x}}_{2}=\frac{-\gamma_{1}a_{2}\sqrt{2gx_{2}}+\left(a_{4}-\gamma_{1}a_{4}\right)\sqrt{2gx_{4}}-\left(a_{1}-\gamma_{1}a_{1}\right)\sqrt{2gx_{1}}}{A_{2}\gamma_{1}},\\ & \;{\dot{x}}_{3}=0,\\ & \;{\dot{x}}_{4}=0. \end{array}$$

The closed-loop system (15) shows that $x_{3}$ and $x_{4}$ will reach the intersection manifold *S* in finite time. □

## 4. Design of the Modulating-Function-Based Backstepping Super-Twisting Controller

Multivariable control of the QTS based on the synthesis of a controller by the equivalent control method is feasible. Nonetheless, the implementation of the first-order sliding modes in the QTS could be detrimental to the plant actuators due to the chattering and the sensitivity to the measurement noise. On the other hand, using the backstepping and modulating functions technique can in fact render the controller chattering-free and robust to nonlinear noise. In this section, a modulating-function-based backstepping super-twisting controller is designed for the first time.

The backstepping technique [28] is a recursive back-deduced Lyapunov-based approach for systems transformable in their parametric-strict-feedback form or their pure parametric feedback form. This technique uses some of the system state variables as “virtual controls” at each step of the algorithm, implementing intermediate control laws to stabilize the system energy [28]. The advantage of applying backstepping control (BSC) is that it avoids the cancellation of the nonlinearities that are useful for controlling the system, easing the control effort. Moreover, the BS technique provides a framework to develop adaptive laws to unknown process parameters [29,30] and calibrate the gains of the online sliding mode controller, to efficiently compensate for parametric uncertainties and disturbances. However, its dependence on the derivatives of the state variables limits its application in plants or processes where these derivatives are not measurable. In general, recent nonlinear SMC and BSC approaches for liquid level control of the QTS [22,23,24,25,31,32] have been proved to be efficient only in conditions where the derivatives of the state variables are available and in the absence of measurement noise.

***Design of the backstepping super-twisting controller (BSSTC) for tank n°4***. The dynamics of the QTS are reduced only by considering the dynamics of the tank n°4. In order to find a suitable STC through the backstepping technique, it is necessary to extend the dynamics of tank n°4 by adding an auxiliary input $w_{1}$ as follows:

(16) $$\begin{array}{cc} & \;{\dot{h}}_{4}=A\sqrt{h_{4}}+B\sqrt{h_{1}}+Cu_{1}+\xi_{4},\\ & \;\dot{u_{1}}=w_{1}, \end{array}$$

where $A=-\frac{a_{4}\sqrt{2g}}{A_{4}}$, $B=\frac{a_{1}\sqrt{2g}}{A_{4}}$, and $C=\frac{\gamma_{1}k_{1}}{A_{4}}$. For the extended subsystem above, the following coordinate change is introduced:

(17) $$\begin{array}{cc} & \;{\dot{x}}_{1}=\frac{A}{2}+\frac{B}{2x_{1}}x_{2}+\frac{C}{2x_{1}}u_{1}+\frac{1}{2x_{1}}\xi_{4},\\ & \;\dot{u_{1}}=w_{1}, \end{array}$$

where $x_{1}=\sqrt{h_{4}}$, $x_{2}=\sqrt{h_{1}}$, and ${\dot{u}}_{1}=w_{1}$ is an auxiliary input. By adding $\dot{u_{1}}=w_{1}$, system (19) is already in pure parametric feedback form [28]. The control objective is to design a continuous controller that regulates the liquid level of tank n°4 at any operating point of the plant, achieving a SOSM ($\sigma_{i}={\dot{\sigma }}_{i}=0\;\forall \;i=1,2$) in finite time over the sliding surface $\sigma_{i}\left(x\right)$.

**Step 1**. Starting with the dynamics of $x_{1}$ in system (17), let us define the new error coordinate,

(18) $$z_{1}=x_{1}-{x_{1}}^{*},$$

where ${x_{1}}^{*}$ is a twice-continuously differentiable reference trajectory. However, if the reference trajectory ${x_{1}}^{*}$ is a step function, ${\dot{x}}_{1}^{*}={\ddot{x}}_{1}^{*}={{\dddot{x}}_{1}}^{*}=0$ holds. So, the derived dynamics of the new coordinate are:

(19) $${\dot{z}}_{1}=\frac{A}{2}+\frac{B}{2x_{1}}x_{2}+\frac{C}{2x_{1}}u_{1}+\frac{1}{2x_{1}}\xi_{4}-{\dot{x}}_{1}^{*}.$$

Let $x_{2}$ be an internal control variable, $\alpha_{1}$ a virtual control law, and $z_{2}=x_{2}-\alpha_{1}$ the error between the actual control variable and the virtual control. Then, the control objective is to design the virtual control law $\alpha_{1}$, such that $z_{1}\to 0$ in finite time. In order to stabilize $\sigma_{1}\to 0$, the following candidate Lyapunov function is introduced:

(20) $$V_{1}=\frac{1}{2}{z_{1}}^{2},$$

and its time derivative:

(21) $${\dot{V}}_{1}=z_{1}{\dot{z}}_{1}.$$

Replacing Equation (21) in (23) yields:

(22) $${\dot{V}}_{1}=z_{1}\left(\frac{A}{2}+\frac{B}{2x_{1}}\alpha_{1}+\frac{C}{2x_{1}}u_{1}+\frac{1}{2x_{1}}\xi_{4}-{\dot{x}}_{1}^{*}\right)+\frac{B}{2x_{1}}z_{2}z_{1}.$$

If ${\dot{z}}_{1}=-c_{1}z_{1}$, where $c_{1}>0$ must hold, then

(23) $${\dot{V}}_{1}=-c1{z_{1}}^{2}+\frac{B}{2x_{1}}z_{2}z_{1}<0,$$

if and only if $z_{2}$ = 0, and $z_{1}$ is locally asymptotically stable. To this end, virtual control law $\alpha_{1}$ is found:

(24) $$\alpha_{1}=-\frac{A}{B}x_{1}-\frac{C}{B}u_{1}-\frac{1}{B}\xi_{4}+\frac{2}{B}\Phi_{1}-\frac{2c_{1}}{B}\Phi_{2},$$

with dynamics:

(25) $${\dot{\alpha }}_{1}=-\frac{A}{B}{\dot{x}}_{1}-\frac{C}{B}{\dot{u}}_{1}-\frac{1}{B}{\dot{\xi }}_{4}+\frac{2}{B}{\dot{\Phi }}_{1}-\frac{2c_{1}}{B}{\dot{\Phi }}_{2},$$

where

(26) $$\begin{array}{cc} & \;\Phi_{1}=x_{1}{\dot{x}}_{1}^{*},\\ & \;\Phi_{2}=x_{1}z_{1},\\ & \;{\dot{\Phi }}_{1}={\dot{x}}_{1}{\dot{x}}_{1}^{*}+x_{1}{\ddot{x}}_{1}^{*},\\ & \;{\dot{\Phi }}_{2}={\dot{x}}_{1}z_{1}+x_{1}{\dot{z}}_{1}. \end{array}$$

**Step 2**. The dynamics of $z_{2}$ are derived such as:

(27) $$\begin{array}{cc} & \;{\dot{z}}_{2}={\dot{x}}_{2}-{\dot{\alpha }}_{1},\\ & \;{\dot{z}}_{2}={\dot{x}}_{2}+\frac{A}{B}{\dot{x}}_{1}+\frac{C}{B}w_{1}+\frac{1}{B}{\dot{\xi }}_{4}-\frac{2}{B}{\dot{\Phi }}_{1}+\frac{2c_{1}}{B}{\dot{\Phi }}_{2}, \end{array}$$

where $w_{1}={\dot{u}}_{1}$. Then, to make $z_{2}\to 0$ to hold in finite time, the following candidate Lyapunov function is designed:

(28) $$V_{2}=V_{1}+\frac{1}{2}{z_{2}}^{2},$$

and its derivative:

(29) $$\begin{array}{cc} & \;{\dot{V}}_{2}={\dot{V}}_{1}+z_{2}{\dot{z}}_{2},\\ & \;{\dot{V}}_{2}=-c_{1}{z_{1}}^{2}+\frac{B}{2x_{1}}z_{2}z_{1}+z_{2}{\dot{z}}_{2},\\ & \;{\dot{V}}_{2}=-c_{1}{z_{1}}^{2}+z_{2}\left(\frac{B}{2x_{1}}z_{1}+{\dot{z}}_{2}\right). \end{array}$$

To stabilize the energy in the system, $\frac{B}{2x_{1}}z_{1}+{\dot{z}}_{2}=\sigma_{1}-c_{2}z_{2}$ must be fulfilled, where $\sigma_{1}=c_{2}z_{2}+{\dot{z}}_{2}$ is the sliding surface, since $z_{1}\to 0$ holds in finite time. So,

(30) $${\dot{V}}_{2}=-c_{1}{z_{1}}^{2}+z_{2}\sigma_{1}-c_{2}{z_{2}}^{2}<0.$$

**Step 3**. The sliding surface,

(31) $$\sigma_{1}=c_{2}z_{2}+{\dot{z}}_{2}+\frac{B}{2}\Phi_{3},$$

with dynamics

(32) $${\dot{\sigma }}_{1}=c_{2}{\dot{z}}_{2}+{\ddot{z}}_{2}+\frac{B}{2}{\dot{\Phi }}_{3},$$

where

(33) $$\begin{array}{cc} & \;{\ddot{z}}_{2}={\ddot{x}}_{2}+\frac{A}{B}{\ddot{x}}_{1}+\frac{C}{B}{\dot{w}}_{1}+\frac{1}{B}{\ddot{\xi }}_{4}-\frac{2}{B}{\ddot{\Phi }}_{1}+\frac{2c_{1}}{B}{\ddot{\Phi }}_{2},\\ & \;\Phi_{3}=\frac{z_{1}}{x_{1}},\\ & \;{\dot{\Phi }}_{3}=\frac{{\dot{z}}_{1}x_{1}-z_{1}{\dot{x}}_{1}}{{x_{1}}^{2}},\\ & \;{\ddot{\Phi }}_{1}={\ddot{x}}_{1}{\dot{x}}_{1}^{*}+{\dot{x}}_{1}{\ddot{x}}_{1}^{*}+{\dot{x}}_{1}{\ddot{x}}_{1}^{*}+x_{1}{{\dddot{x}}_{1}}^{*},\\ & \;{\ddot{\Phi }}_{2}={\ddot{x}}_{1}z_{1}+{\dot{x}}_{1}{\dot{z}}_{1}+{\dot{x}}_{1}{\dot{z}}_{1}+x_{1}{\ddot{z}}_{1}, \end{array}$$

must be designed, such that ${\dot{V}}_{2}$ decreases its energy in a finite time. Then, the following candidate Lyapunov function is introduced:

(34) $$V_{3}=V_{2}+\frac{1}{2}{\sigma_{1}}^{2},$$

with time derivative:

(35) $$\begin{array}{cc} & \;{\dot{V}}_{3}={\dot{V}}_{2}+\sigma_{1}{\dot{\sigma }}_{1},\\ & \;{\dot{V}}_{3}=-c_{1}{z_{1}}^{2}+z_{2}\sigma_{1}-c_{2}{z_{2}}^{2}+\sigma_{1}{\dot{\sigma }}_{1},\\ & \;{\dot{V}}_{3}=-c_{1}{z_{1}}^{2}-c_{2}{z_{2}}^{2}+\sigma_{1}\left(z_{2}+{\dot{\sigma }}_{1}\right). \end{array}$$

Thus, to obtain

(36) $${\dot{V}}_{3}=-c_{1}{z_{1}}^{2}-c_{2}{z_{2}}^{2}-c_{3}{\sigma_{1}}^{2}<0,$$

or

(37) $${\dot{V}}_{3}=-\sum_{i=1}^{2}c_{i}{z_{i}}^{2}-c_{3}{\sigma_{1}}^{2}<0,$$

the following relationship must hold:

(38) $$z_{2}+{\dot{\sigma }}_{1}=-c_{3}\sigma_{1},$$

where $c_{3}>0$. Replacing Equations (31)–(33) in (38) yields:

(39) $$z_{2}+c_{2}{\dot{z}}_{2}+{\ddot{x}}_{2}+\frac{A}{B}{\ddot{x}}_{1}+\frac{C}{B}{\dot{w}}_{1}+\frac{1}{B}{\ddot{\xi }}_{4}-\frac{2}{B}{\ddot{\Phi }}_{1}+\frac{2c_{1}}{B}{\ddot{\Phi }}_{2}+\frac{B}{2}{\dot{\Phi }}_{3}=-c_{3}\left(c_{2}z_{2}+{\dot{z}}_{2}\right)-c_{3}\frac{B}{2}\Phi_{3}.$$

Later, term ${\dot{w}}_{1}$ is isolated:

(40) $$\begin{array}{cc} {\dot{w}}_{1}= & \;\frac{B}{C}\left(-z_{2}-c_{2}{\dot{z}}_{2}-{\ddot{x}}_{2}-\frac{A}{B}{\ddot{x}}_{1}+\frac{2}{B}{\ddot{\Phi }}_{1}-\frac{2c_{1}}{B}{\ddot{\Phi }}_{2}-\frac{B}{2}{\dot{\Phi }}_{3}-c_{3}c_{2}z_{2}-c_{3}{\dot{z}}_{2}-c_{3}\frac{B}{2}\Phi_{3}\right)-\frac{1}{C}{\ddot{\xi }}_{4}. \end{array}$$

Then, by performing the double integration of term ${\dot{w}}_{1}$, the actual control $u_{1}$ is obtained:

(41) $$\begin{array}{cc} & \;u_{1}=\int_{0}^{t}\int_{0}^{t}{\dot{w}}_{1}d\tau d\tau -\frac{1}{C}\xi_{4}-\lambda_{1}{|\sigma_{1}|}^{1/2}sign\left(\sigma_{1}\right)-\int_{0}^{t}\lambda_{2}sign\left(\sigma_{1}\right)d\tau . \end{array}$$

The super-twisting terms $\lambda_{1}{|\sigma_{1}|}^{1/2}sign\left(\sigma_{1}\right)$ and $\int_{0}^{t}\lambda_{2}sign\left(\sigma_{1}\right)d\tau$, where $\lambda_{1}=1.5{\left|\Delta \right|}^{1/2},$ $\lambda_{2}=1.1\left|\Delta \right|>0$, and |Δ| is an upper-bound of the expected disturbance to the system, are added to provide robust compensation for the disturbances to and uncertainties in the system. Replacing (40) in (41), the variable dependencies of the BSSMC control law are taken into account:

(42) $$\begin{array}{cc} u_{1}= & \;\int_{0}^{t}\int_{0}^{t}(\frac{B}{C}(-z_{2}-c_{2}{\dot{z}}_{2}-{\ddot{x}}_{2}-\frac{A}{B}{\ddot{x}}_{1}+\frac{2}{B}\ddot{\Phi_{1}}-\frac{2c_{1}}{B}{\ddot{\Phi }}_{2}-\frac{B}{2}{\dot{\Phi }}_{3}-c_{3}c_{2}z_{2}-c_{3}{\dot{z}}_{2}\\ & \;-c_{3}\frac{B}{2}\Phi_{3}))d\tau d\tau -\lambda_{1}{|\sigma_{1}|}^{1/2}sign\left(\sigma_{1}\right)-\int_{0}^{t}\lambda_{2}sign\left(\sigma_{1}\right)d\tau . \end{array}$$

It should be noted that the same procedure is carried out to obtain the control law $u_{2}$. It is important to state, after obtaining the control law $u_{1}$, that for the classical backstepping technique only two steps would be needed to obtain an adequate control law. However, by taking an additional step in the algorithm, the control law is integrated twice, giving additional integral action for the removal of the rate of change of the error variables at the steady state, as well as giving increased robustness against the disturbances and parameter uncertainties. Since the control law $u_{1}$ depends on unmeasurable derivatives ${\dot{x}}_{1}$, ${\dot{x}}_{2}$, ${\ddot{x}}_{1}$, and ${\ddot{x}}_{2}$, the state-of-the-art approach would force the implementation of differentiators, such as in [21,23], that would amplify the noise coming from the measured liquid levels $y_{1}$ and $y_{4}$. For this, the technique of modulating functions is proposed to obtain the sliding surfaces $\sigma_{i}\left(x\right)$ that do not depend on the derivatives of the state variables and are noise-free.

The modulating functions (MF) technique performs integral transformations to compute the derivative-free algebraic relations between the inputs and outputs of a system, which allows the estimation of the internal states, unknown parameters, and fault detection with the advantage of not relying on the derivatives of the state variables and filtering noise while the modulation operation is carried out. To understand the mathematical background of the MF technique, the following definitions are presented.

**Definition 3**  (Total Modulating Function [33])**.** *Consider a sufficiently smooth function R×R→R, with partial derivatives as*

(43) $$\varphi^{\left(i\right)}\left(t,t_{1}\right):={\left.\frac{\partial^{i}\varphi }{\partial \tau^{i}}\left(\tau ,t_{1}\right)\right|}_{\tau =t}.$$

*Then, function φ is called a modulating function of order* k *, if there exists $t_{0}<t_{1}$, such that*

(44) $$\varphi^{\left(i\right)}\left(t_{0},t_{1}\right)\cdot \varphi^{\left(i\right)}\left(t_{1},t_{1}\right)=0,\forall i\;=\;0,1,\ldots ,n-1.$$

*A modulating function whose boundaries satisfy $\varphi^{\left(i\right)}\left(t_{0},t_{1}\right)=\varphi^{\left(i\right)}\left(t_{1},t_{1}\right)=0$ is called a total modulating function.*

**Definition 4**  (Modulation Functional [34])**.** *The modulation functional is defined as:*

(45) $$M\left[h\right]={\langle h,\varphi \rangle }_{\Omega ,I}=\int_{\Omega }\int_{0}^{t}h\left(x,\tau +t-T\right)\varphi \left(x,\tau \right)d\tau dx,$$

*where* h *: $\Omega \times R_{0}^{+}\to R$ and φ:Ω×I→R represent the signal to be modulated and the modulating function, respectively, spatially defined on the* n*-dimensional rectangular region $\Omega :=x\in R^{n}:0<x_{i}<L_{i},i=1,2,\ldots ,n$ and temporally on the moving time horizon I=[t−T,t] of length T>0. Moreover, if the integration concerns only the spatial or temporal variable, the inner product notations ${\langle h,\varphi \rangle }_{\Omega }$ or ${\langle h,\varphi \rangle }_{I}$ are used, respectively.*

When implementing the MFs for filtering, an FIR filter with a modulating receding horizon can be realized, integrating only in the time dimension as follows:

(46) $$\begin{array}{cc} & \;M^{i}\left[h\right]={\left(-1\right)}^{i}\int_{t-T}^{t}\phi^{i}\left(t-\tau +T\right)h\left(\tau \right)\;d\tau ,\\ & \;M^{i}\left[h\right]≅\;{\left(-1\right)}^{i}T_{s}\sum_{k=0}^{N}W_{k}\phi^{i}\left(kT_{s}\right)h\left(\left(l-N+k\right)T_{s}\right), \end{array}$$

and we define it in its matrix form

(47) $$M^{i}\left[h\right]=K_{MF}^{i}\left[\begin{array}{c} h(l-N)\\ .\\ .\\ .\\ h(l-1)\\ h(l) \end{array}\right],$$

where

(48) $$K_{MF}^{i}={\left(-1\right)}^{i}T_{s}{\left[\begin{array}{c} W_{0}\varphi^{\left(i\right)}\left(0\right)\\ .\\ .\\ .\\ W_{N-1}\varphi^{\left(i\right)}\left(\left(N-1\right)T_{s}\right)\\ W_{N}\varphi^{\left(i\right)}\left(NT_{s}\right) \end{array}\right]}^{T}$$

is an i-dimension vector of modulating gains sampled with $t\;=\;lT_{s},\;l\;\in \;N$.

**Lemma 1**  (Shift of derivatives property)**.** *Using integration by parts, a shift of derivatives can be attained as follows:*

(49) $$\begin{array}{cc} & \;M^{0}\left[h^{\left(n\right)}\right]=\int_{t-T}^{t}\varphi \left(t-\tau +T\right)h^{\left(n\right)}\left(\tau \right)\;d\tau ,\\ & \;M^{0}\left[h^{\left(n\right)}\right]=M^{n}\left[h\right]≅\;{\left(-1\right)}^{n}\int_{t-T}^{t}\varphi^{\left(n\right)}\left(t-\tau +T\right)h\left(\tau \right)\;d\tau . \end{array}$$

*After presenting these definitions, the task is to modulate the error coordinates $z_{1}$ and $z_{2}$, the virtual control law $\alpha_{1}$, and the sliding surface $\sigma_{1}$, such that control $u_{1}$ is resilient to noise and does not depend on the derivatives of the system’s state variables. For this, the error coordinates $z_{1}$,*

(50) $$\begin{array}{cc} & \;M^{0}\left[z_{1}\right]=M^{0}\left[x_{1}-{x_{1}}^{*}\right]\\ & \;M^{0}\left[z_{1}\right]=M^{0}\left[\sqrt{h_{4}}\right]-M^{0}\left[\sqrt{{h_{4}}^{*}}\right],\\ & \; \end{array}$$

*and $z_{2}$,*

(51) $$\begin{array}{cc} & \;M^{0}\left[z_{2}\right]=M^{0}\left[x_{2}-\alpha_{1}\right]\\ & \;M^{0}\left[z_{2}\right]=M^{0}\left[\sqrt{y_{1}}\right]+\frac{A}{B}M^{0}\left[x_{1}\right]+\frac{C}{B}M^{0}\left[u_{1}\right]-\frac{2}{B}M^{0}\left[\Phi_{1}\right]+\frac{2c_{1}}{B}M^{0}\left[\Phi_{2}\right], \end{array}$$

*are modulated over a prescribed modulation receding horizon. The nonlinearities $\sqrt{h_{1}}$, $\sqrt{h_{4}}$, $\Phi_{1}$, and $\Phi_{2}$ in Equations (52) and (53) cannot be directly modulated. However, if $h_{1}$ and $h_{4}$ are replaced by measurements $y_{1}$ and $y_{4}$, respectively, and ${h_{4}}^{*}$ and ${\dot{h}}_{4}^{*}$ are known trajectories, then these nonlinear terms can be computed numerically and modulated on each iteration. Moreover, after modulating $z_{2}$, the following modulated virtual control law $\alpha_{1}$ is obtained:*

(52) $$\begin{array}{c} M^{0}\left[\alpha_{1}\right]=-\frac{A}{B}M^{0}\left[\sqrt{y_{4}}\right]-\frac{C}{B}M^{0}\left[u_{1}\right]+\frac{1}{B}M^{0}\left[\Phi_{1}\right]-\frac{2c_{1}}{B}M^{0}\left[\Phi_{2}\right], \end{array}$$

*with modulated dynamics:*

(53) $$\begin{array}{cc} & \;M^{0}\left[{\dot{\alpha }}_{1}\right]=M^{0}\left[-\frac{A}{B}{\dot{x}}_{1}-\frac{C}{B}{\dot{u}}_{1}+\frac{2}{B}{\dot{\Phi }}_{1}-\frac{2c_{1}}{B}{\dot{\Phi }}_{2}\right],\\ & \;M^{1}\left[\alpha_{1}\right]=-\frac{A}{B}M^{1}\left[\sqrt{y_{4}}\right]-\frac{C}{B}M^{1}\left[u_{1}\right]+\frac{2}{B}M^{1}\left[\Phi_{1}\right]-\frac{2c_{1}}{B}M^{1}\left[\Phi_{2}\right]. \end{array}$$

*Finally, the sliding surface is modulated as follows:*

(54) $$\begin{array}{cc} & \;M^{0}\left[\sigma_{1}\right]=M^{0}\left[c_{2}z_{2}+{\dot{z}}_{2}+\frac{B}{2}\Phi_{3}\right],\\ & \;M^{0}\left[\sigma_{1}\right]=c_{2}M^{0}\left[z_{2}\right]+M^{1}\left[z_{2}\right]+\frac{B}{2}M^{0}\left[\Phi_{3}\right], \end{array}$$

*with dynamics:*

(55) $$\begin{array}{cc} & \;M^{0}\left[{\dot{\sigma }}_{1}\right]=M^{0}\left[c_{2}{\dot{z}}_{2}+{\ddot{z}}_{2}+\frac{B}{2}{\dot{\Phi }}_{3}\right],\\ & \;M^{1}\left[\sigma_{1}\right]=c_{2}M^{1}\left[z_{2}\right]+M^{2}\left[z_{2}\right]+\frac{B}{2}M^{1}\left[\Phi_{3}\right]. \end{array}$$

**Figure 3:**
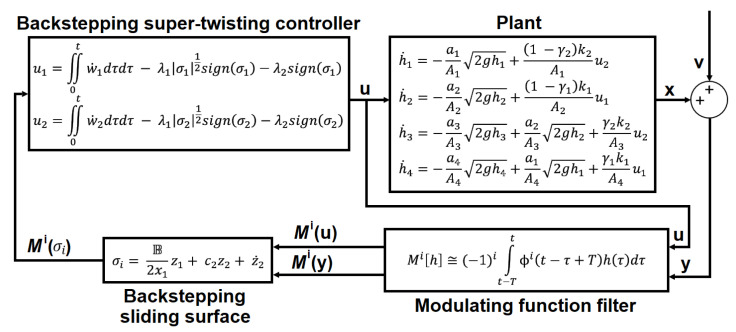
Block diagram of the modulating-function-based backstepping super-twisting control system for a QTS.

*With the modulating functions technique, all the virtual controls of $u_{1}$ have derivative-free input–output algebraic relations. Even though $u_{1}$ depends on the noise-measured state variables $y_{1}$ and $y_{4}$, the MF technique filters noise when performing integration of these signals. Figure 3 shows the block diagram of the modulating-function-based backstepping super-twisting control system for a QTS.*

## 5. Results and Discussion

### 5.1. Multivariable Sliding Mode Control of the Liquid Levels in Tanks n°3 and n°4

The control objective is to regulate the liquid levels of tank n°3 and n°4 at different operating points with a nonlinear controller. To this end, the performance of a first-degree sliding mode controller synthesized with the equivalent control method and a BSSTC were compared in the absence of measurement noise and disturbances, for a total simulation time $T_{sim}$ = 1000 s and sample time $T_{s}=0.01$ s. The model parameters and liquid level operating points, the latter modeled as step inputs to the system, are shown in Table 1 and Table 2, respectively. The lower and upper saturation thresholds for the pumps control efforts were set at 10% and 60%, respectively. The design specifications of the BSSTC are shown in Table 3.

**Figure 4:**
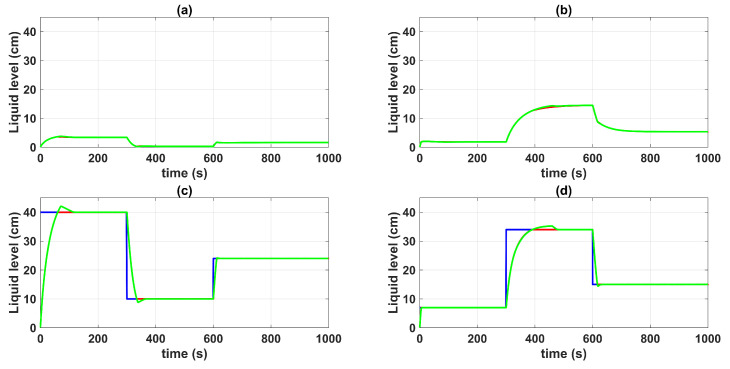
Control system time responses of all tanks: (**a**) tank n°1; (**b**) tank n°2; (**c**) tank n°3; (**d**) tank n°4; QTS with SMC (red line); QTS with BSSTC (green line); set point (blue line).

Figure 4 shows the control system time responses of all tanks at the operating points described in Table 2, where the liquid levels of tanks n°3 and n°4 were satisfactorily controlled by both the first-degree SMC and by the BSSTC. In addition, since the system valves were calibrated for the minimum phase response, the liquid levels in tanks n°1 and n°2 remained at safe operating points without overflow.

**Figure 5:**
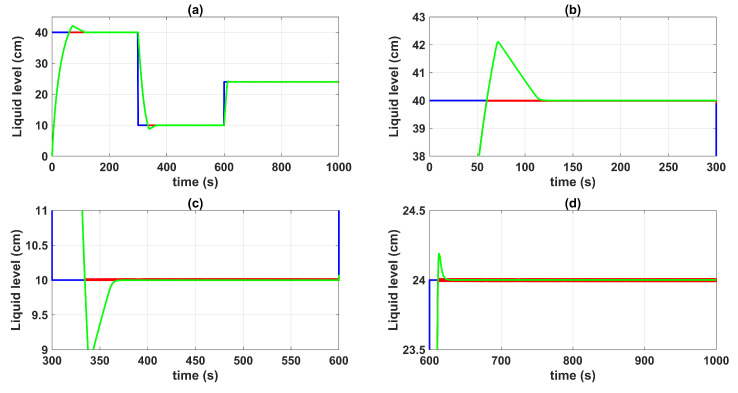
Control system time responses of tank n°3: (**a**) t = [0 1000] s; (**b**) t = [0 300] s; (**c**) t = [300 600] s; (**d**) t = [600 1000] s; QTS with SMC (red line); QTS with BSSTC (green line); set point (blue line).

**Figure 6:**
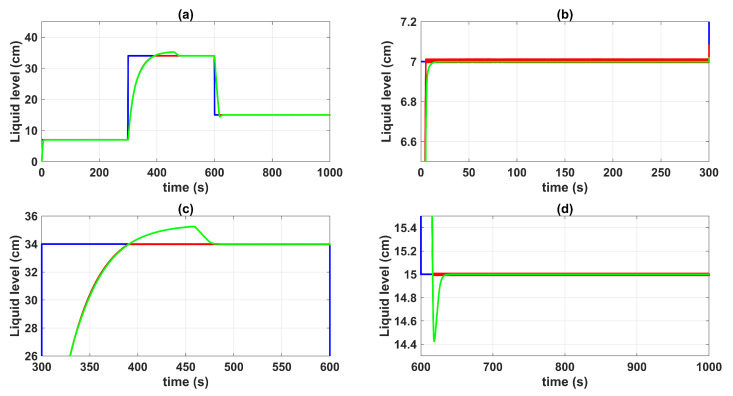
Control system time responses of tank n°4: (**a**) t = [0 1000] s; (**b**) t = [0 300] s; (**c**) t = [300 600] s; (**d**) t = [600 1000] s; QTS with SMC (red line); QTS with BSSTC (green line); set point (blue line).

**Figure 7:**
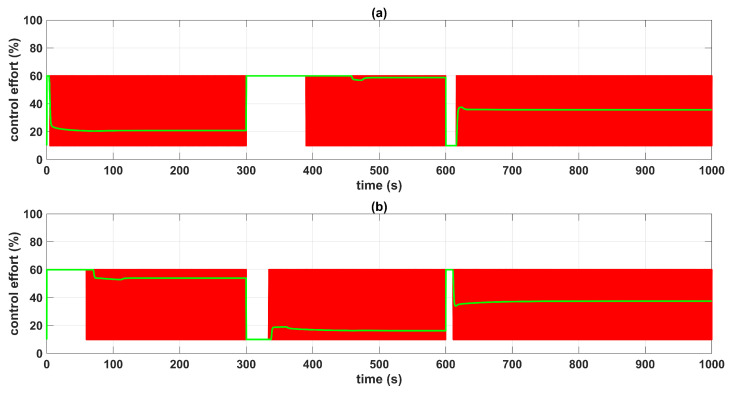
Control efforts $u_{1}$ and $u_{2}$: (**a**) control effort $u_{1}$; (**b**) control effort $u_{2}$; QTS with SMC (red line); QTS with BSSTC (green line).

Figure 5 and Figure 6 show the control system time responses of tanks n°3 and n°4 in the different time intervals, where the SMC had a faster settling time than the BSSTC. The drawback of using the SMC for this specific application is seen in Figure 7, where the control efforts $u_{1}$ and $u_{2}$ of the SMC exhibited a considerable energy effort with undesirable chattering that oscillated from 10% to 60% of the pumping effort. This condition would be detrimental to the pumps actuators. On the other hand, the BSSTC did not present chattering in the absence of noise, and the energy effort was much lower than the SMC.

**Figure 8:**
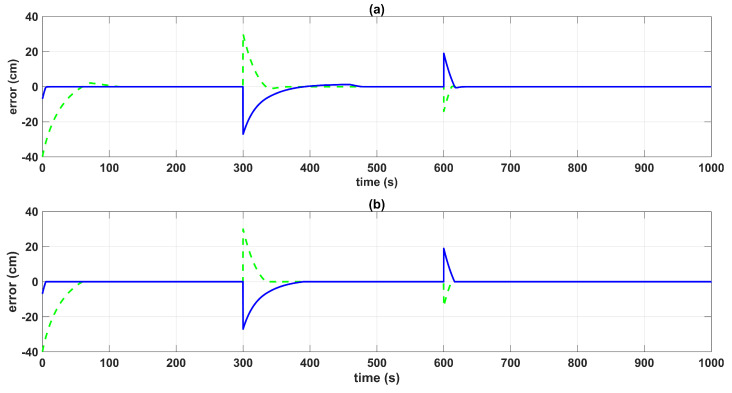
Control system errors: (**a**) QTS with BSSTC; (**b**) QTS with SMC; error $h_{3}$-$h_{3}*$ (green dotted line); error $h_{4}$-$h_{4}*$ (blue line).

Figure 8 shows the errors between the actual and the desired liquid level over time, where it is noted that the error with the SMC converged faster than with the BSSTC. Moreover, Table 4 shows the results related to the following performance indices: the integral time absolute error (ITAE), integral absolute error (IAE), and the integral square error (ISE), where the SMC obtained better scores since it had a faster convergence, without overshoot, compared to the BSSTC, which showed a slower response with overshoot in some intervals of the trajectory. This result is especially important as it shows that although the SMC obtains slightly better dynamic error scores, it does so at the expense of more energy used in the control effort and the introduction of chattering.

Although, the BSSTC would work in conditions where the derivatives of the system state variables were accessible, and there was no measurement noise, this is not the case in most industrial applications, where sensors are not available due to budget constraints or they are not physically realizable. For example, for this plant, the velocity and acceleration of the liquid level could not be directly measured.

### 5.2. Modulating-Function-Based Backstepping Super-Twisting Control of Liquid Levels in Tanks n°3 and n°4

**Figure 9:**
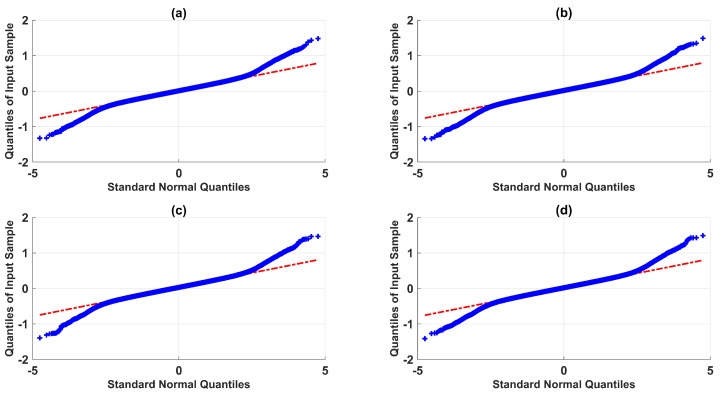
Gaussian mixture quantile–quantile plot: (**a**) level transmitter $LT_{1}$; (**b**) level transmitter $LT_{2}$; (**c**) level transmitter $LT_{3}$; (**d**) level transmitter $LT_{4}$.

**Figure 10:**
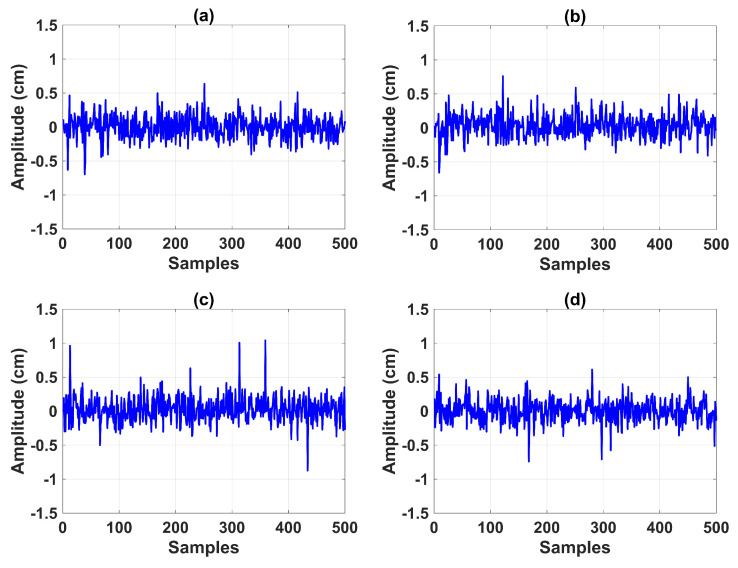
Noise amplitude: (**a**) level transmitter $LT_{1}$; (**b**) level transmitter $LT_{2}$; (**c**) level transmitter $LT_{3}$; (**d**) level transmitter $LT_{4}$.

For this end, the goal was to test the robustness of the modulating-function-based BSSTC against nonlinear measurement noise. Noise with a mixture of Gaussian distributions was generated for the level transmitters $LT_{1}$, $LT_{2}$, $LT_{3}$, and $LT_{4}$, according to the mixture probability $p\left(x\right)=\left(1-\epsilon \right)p_{g1}+\epsilon p_{g2}$, where ϵ is the probability for distribution $p_{g2}\;N\left(0,{\left(\sigma_{2}\right)}^{2}\right)$, and $\sigma_{1}$ and $\sigma_{2}$ are the standard deviations. Table 5 shows the specifications of the Gaussian mixture model (GMM). In Figure 9 and Figure 10, the quantile–quantile plots (QQ plots) and sample vs. amplitude plots are shown, respectively, where it is evident that the GMM exhibited nonlinear characteristics.

**Figure 11:**
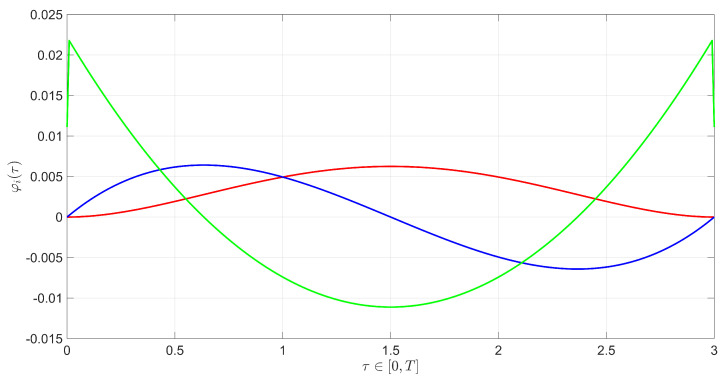
Modulating functions: modulating function kernel (green line); modulating function kernel first derivative (blue line); modulating function kernel second derivative (red line).

In Figure 3, the modulating function filter was synthesized with a modulation function kernel order of one and two kernel derivatives, with a polynomial waveform type, a time horizon interval of 3.00 s, and a sample time of 0.01 s. The polynommial waveforms of the kernel and its two derivatives are shown in Figure 11. It received the measurement vector *y* and the control vector *u* as inputs, and sent the modulated vectors $M^{i}\left[u\right]$ and $M^{i}\left[y\right]$ to the backstepping sliding surface block. The backstepping sliding surface block generated the modulated sliding surfaces $\sigma_{1}$ and $\sigma_{2}$. Finally, the backstepping super-twisting control yielded the modulated virtual control $w_{i}$ and output a modulated backstepping control signal along the super-twisting terms for the plant.

For this result, the performance of the MF-BSSTC in the presence of measurement noise was tested, setting up the gains according to Table 6.

**Figure 12:**
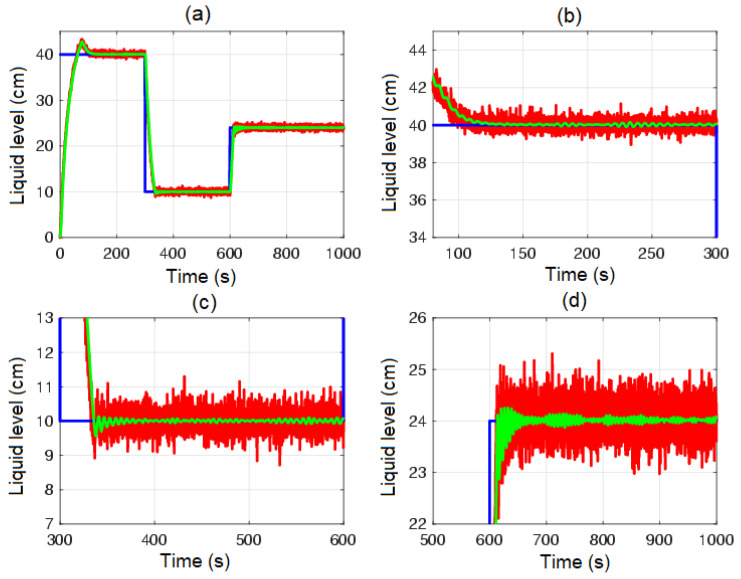
Control system time responses of tank n°3: (**a**) t = [0 1000] s; (**b**) t = [0 300] s; (**c**) t = [300 600] s; (**d**) t = [600 1000] s; control system time responses with measurement noise (red line); control system time responses with MF-BSSTC (green line); set point (blue line).

**Figure 13:**
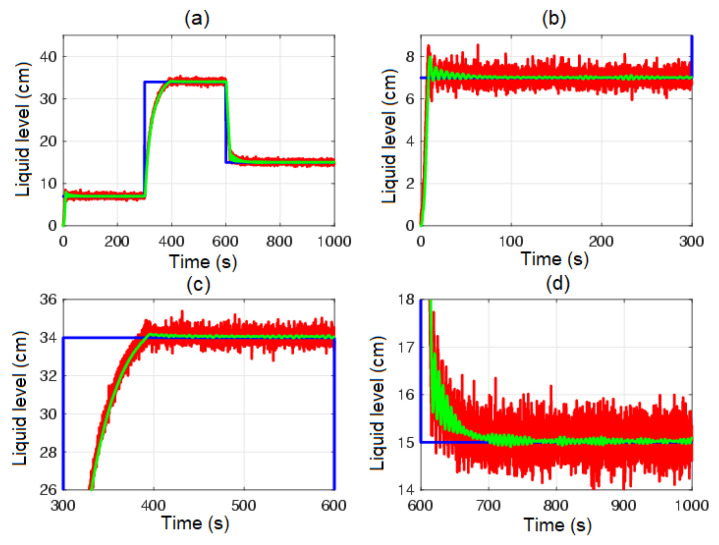
Control system time responses of tank n°4: (**a**) t = [0 1000] s; (**b**) t = [0 300] s; (**c**) t = [300 600] s; (**d**) t = [600 1000] s; control system time responses with measurement noise (red line); control system time responses with MF-BSSTC (green line); set point (blue line).

In Figure 12 and Figure 13, the closed-loop time response of the QTS control system with the MF-BSSTC (green line) is plotted against the closed-loop time response of the system with measurement noise (red line). It is remarkable that the MF technique adequately filtered out the noise by modulating all the virtual controls of the backstepping algorithm, rendering it free from the derivatives of the system state variables. Nonetheless, the MF technique generated a delay of 1.5 s. The method to compensate for this delay, either a Smith predictor or any other delay-compensation technique, is outside the scope of this paper.

**Figure 14:**
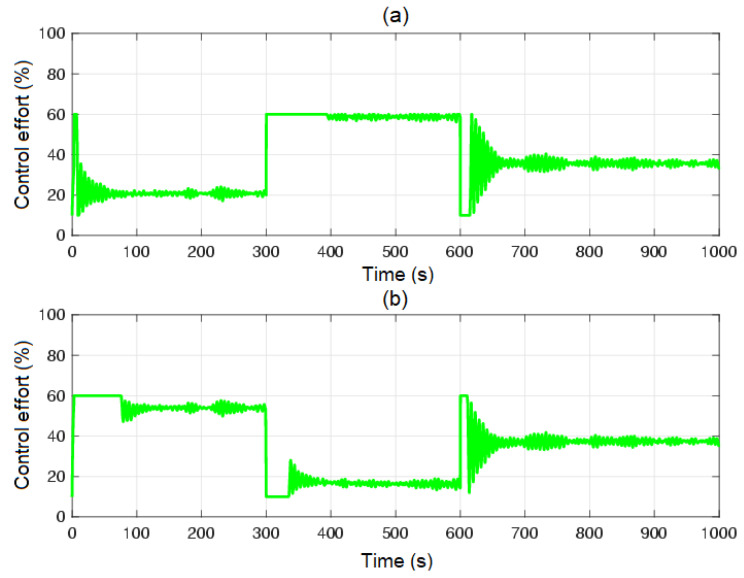
Control efforts $u_{1}$ and $u_{2}$: (**a**) control effort $u_{1}$; (**b**) control effort $u_{2}$; MF-BSSTC (green line).

The controller gains were set to minimize the amplitude of the oscillations in the closed-loop response of the system. Since the MF-BSSTC was tested with non-Gaussian measurement noise, which is a mixture of two different Gaussian distributions, the MF-BSSTC effectively filtered both distributions, but a minimal amplitude variation over time was still visible when the Gaussian mixture changed from one distribution to another. Figure 14 shows the control efforts $u_{1}$ and $u_{2}$, which exhibited minimum oscillations at steady state that were caused by the varying amplitude of the filtered noise. If the gains were increased to improve the settling time and robustness of the response, then the oscillations would increase, and with this, the control effort itself would show greater oscillations. Then, the MF-BSSTC must be synthesized based on the required closed-loop behavior and in terms of a relationship between the noise filtering capability and the desired robustness of the controller.

**Figure 15:**
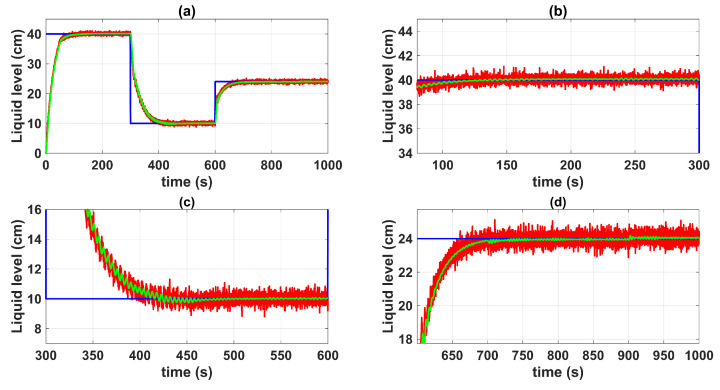
Control system time responses of tank n°3 in the presence of a disturbance to the system: (**a**) t = [0 1000] s; (**b**) t = [0 300] s; (**c**) t = [300 600] s; (**d**) t = [600 1000] s; control system time responses with measurement noise (red line); control system time responses with MF-BSSTC (green line); set point (blue line).

**Figure 16:**
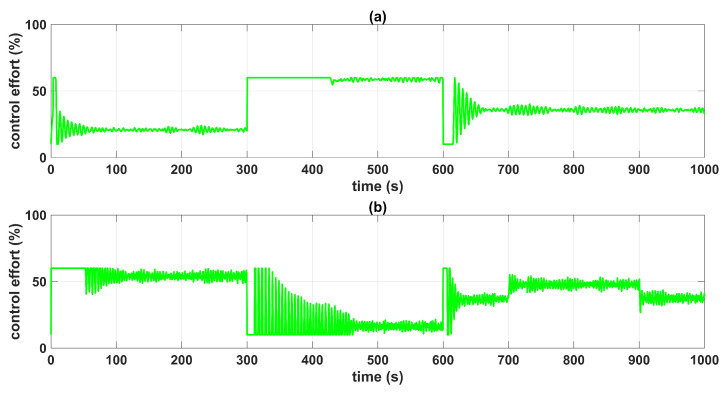
Control efforts $u_{1}$ and $u_{2}$ in the presence of a disturbance to the system: (**a**) control effort $u_{1}$; (**b**) control effort $u_{2}$; MF-BSSTC (green line).

To test the robustness of the controller in the presence of measurement noise, a disturbance $\xi_{4}=-300\;m^{3}/s$ was applied to the system in the time interval *t* = [700 900] s, corresponding to a loss of flow in pump n°2. Then, the MF-BSSTC gains were increased to $\lambda_{1}=29.24$ and $\lambda_{2}=418$, for |Δ|=380, so that the controller offered more robustness. Figure 15 shows that the controller was almost insensitive to the disturbance. Moreover, this robustness came with the chattering of the control signal $u_{2}$ at time interval *t* = [310 461] s, as shown in Figure 16. At the mentioned time interval, there was an overestimation of the controller gains, set as fixed for the worst-case disturbance. Perhaps, this high-gain condition of the controller could be solved by adapting the gains online.

### 5.3. Comparison with Other Controllers

The MF-BSSTC was compared to two of the most used controllers with the QTS, the decoupled PI controller [12] and the model predictive controller (MPC) [15].

A disturbance $\xi_{4}=-300\;m^{3}/s$ at time interval *t* = [600 1000] s was introduced to the system, corresponding to the flow loss in pump n°2. The MF-BSSTC gains for subcontrollers n°1 and n°2 were $c_{1}=1.65$, $c_{2}=1.05$, $c_{3}=1.0$, $\lambda_{1}=29.24$, and $\lambda_{2}=418$ and $c_{1}=1.70$, $c_{2}=1.05$, $c_{3}=1.0$, $\lambda_{1}=29.24$, and $\lambda_{2}=418$, respectively. The decoupled PI controller was synthesized using the MATLAB PID tuner app, with gains $K_{p1}=19.01$ and $k_{i1}=0.74$, and $K_{p1}=26.91$ and $k_{i1}=1.18$, respectively, which maximized the robust transient behavior. On the other hand, the MPC was set with a prediction horizon $H_{p}=200$ and a control horizon $H_{c}=200$. Moreover, both linear controllers were linearized with respect to the operating point ($h_{o3}=12.4$, $h_{o4}=12.7$).

**Figure 17:**
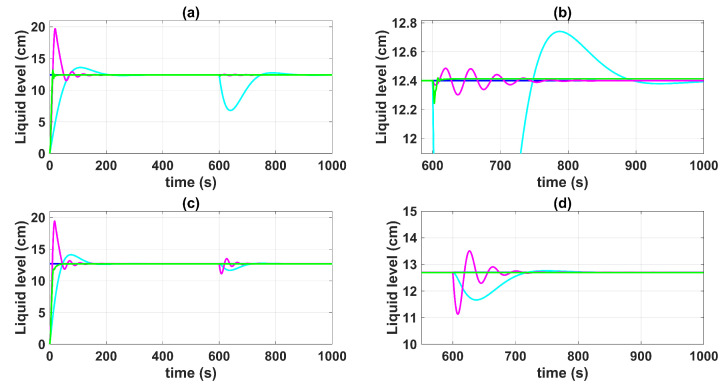
Control system time responses of tank n°3 in the presence of a disturbance to the system: (**a**) t = [0 1000] s; (**b**) t = [0 300] s; (**c**) t = [300 600] s; (**d**) t = [600 1000] s; control system time responses with measurement noise (red line); control system time responses with MF-BSSTC (green line); control system time responses with decoupled PI (cyan line); control system time responses with MPC (magenta line); set point (blue line).

**Figure 18:**
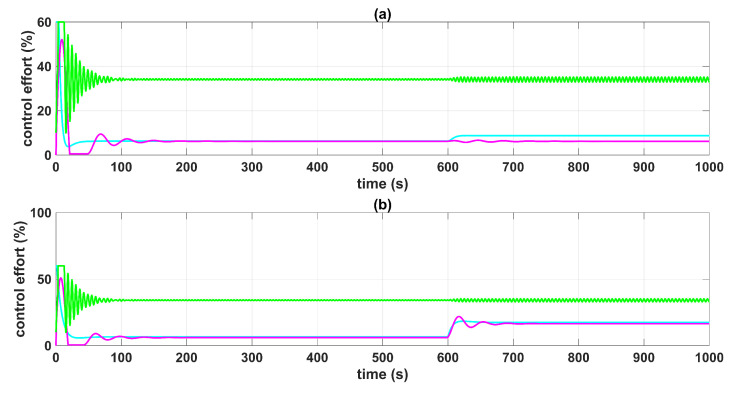
Control efforts $u_{1}$ and $u_{2}$ in the presence of a disturbance to the system: (**a**) control effort $u_{1}$; (**b**) control effort $u_{2}$; MF-BSSTC (green line); decoupled PI (cyan line); MPC (magenta line).

Figure 17 shows that the MF-BSSTC had more robustness to disturbances and a faster response than the decoupled PI and MPC controllers. When disturbed, the PI controller and MPC showed strong set-point deviations from the set point, whereas the MF-BSSTC showed more insensitivity to the disturbance. As shown in Figure 18, the control efforts of the PI and MPC were lower than 10% for all the time responses, indicating that their robustness and speed of convergence were related to this low energy consumption.

Table 7 shows that the best results related to the ITAE, IAE, and ISE criteria were obtained with the MF-BSSTC. The PI and MPC controllers obtained high ITAE and ISE values for subcontrollers $u_{1}$ due to their underdamped responses to the disturbance. Even though they converged to the desired liquid level, their transient responses were very slow.

Regarding the mean computational cost, it was calculated based on the time it took to execute the loop controls. For instance, the mean computational efforts of the MF-BSSTC, PI, and MPC, for a total of 100,000 iterations, were $1.1685\times 10^{-4}$ s, $3.9600\times 10^{-4}$ s, and $2.8959\times 10^{-5}$ s, respectively. Even though the MF-BSSTC had memory requirements related to storing measured samples during the modulating receding horizon, its computational cost was lower than that of the PI controller. In addition, the MPC achieved a lower computational cost.

## 6. Conclusions

In this paper, it has been proved that it is feasible to adopt a nonlinear approach to the QTS liquid level control problem, using a multivariable sliding mode controller. For this, a first-degree SMC and a BSSTC were proposed based on the design of a multivariable sliding surface through the equivalent control method and backstepping virtual controls, respectively.

The results in the absence of noise showed that the liquid level control can be attained at any operating point without the need to linearize the system over a specific operating point. Although, the first-degree SMC exhibited a faster and more robust response, it introduced chattering into the control signal that was detrimental to the actuators of the plant.

On the other hand, the BSSTC showed almost no chattering at the expense of a slower settling time. In addition, a method to make the BSSTC derivative-free and noise-immune based on the modulation of the backstepping virtual controls was proposed, demonstrating that it is possible to use the backstepping algorithm and sliding modes in the presence of non-Gaussian noise through the modulating functions technique at the expense of introducing a time delay equal to half the receding horizon used.

Finally, with respect to future work, we will try to extend the current results considering the time delay in the dynamic behavior of the QTS and the conditions in which this system operates in non-minimum phase mode, as well as leakages and actuator failures.

## Figures and Tables

**Table 1:** QTS parameters.

Parameter	Units	Value
$A_{1}$, $A_{2}$, $A_{3}$, $A_{4}$	cm${}^{2}$	706.85
$a_{1}$, $a_{2}$, $a_{3}$, $a_{4}$	cm${}^{2}$	1.89, 1.89, 5.39, 5.39
*g*	cm/s${}^{2}$	981
$\gamma_{1}$, $\gamma_{2}$	unitless	0.80, 0.90
$k_{1}$, $k_{2}$	unitless	1.00
${u_{1}}_{max}$, ${u_{1}}_{max}$	cm${}^{3}$/s	2863
$d_{1}$, $d_{2}$, $d_{3}$, $d_{4}$	cm	30
${h_{1}}_{max}$, ${h_{2}}_{max}$, ${h_{3}}_{max}$, ${h_{4}}_{max}$	cm	45

**Table 2:** Liquid level operating points.

Operating Point	Time Span	Units	Value
${h_{3}}^{*}$, ${h_{4}}^{*}$	$T_{sim}=\left[\begin{array}{cc} 0 & 300 \end{array}\right]$s	cm	40, 7
${h_{3}}^{*}$, ${h_{4}}^{*}$	$T_{sim}=\left[\begin{array}{cc} 300 & 600 \end{array}\right]$s	cm	10, 34
${h_{3}}^{*}$, ${h_{4}}^{*}$	$T_{sim}=\left[\begin{array}{cc} 600 & 1000 \end{array}\right]$s	cm	24, 15

**Table 3:** The design specifications of the BSSTC for simulation in the absence of measurement noise.

Subcontroller	Parameter	Value (Dimensionless)
$h_{3}$	$c_{1}$, $c_{2}$, $c_{3}$, |Δ|, $\lambda_{1}$, $\lambda_{2}$	1.50, 1.20, 1.00, 1.00, 1.50, 1.10
$h_{4}$	$c_{1}$, $c_{2}$, $c_{3}$, |Δ|, $\lambda_{1}$, $\lambda_{2}$	2.50, 1.50, 1.00, 1.00, 1.50, 1.10

**Table 4:** Performance indices.

Controller	Subcontroller	ITAE (cm)	IAE (cm)	ISE (cm${}^{2}$)
SMC	$h_{3}$	1813.8	1267.9	2525.7
	$h_{4}$	2864.3	782.9	1028.9
BSSTC	$h_{3}$	1919.5	1346.0	2549.2
	$h_{4}$	3203.0	861.1	1050.3

**Table 5:** Gaussian mixture model.

Distr.	Parameters	Units	Values
$p_{g_{1}}$	Mean $\mu_{h_{1}}$, $\mu_{h_{2}}$, $\mu_{h_{3}}$, $\mu_{h_{4}}$	cm	0.01, 0.02, 0.03, 0.02
	Standard deviation $\sigma_{h_{1}}$, $\sigma_{h_{2}}$, $\sigma_{h_{3}}$, $\sigma_{h_{4}}$	cm	0.15
	Probability (1 − ϵ)	unitless	0.95
$p_{g_{2}}$	Mean $\mu_{h_{1}}$, $\mu_{h_{2}}$, $\mu_{h_{3}}$, $\mu_{h_{4}}$	cm	0.05, 0.04, 0.07, 0.04
	Standard deviation $\sigma_{h_{1}}$, $\sigma_{h_{2}}$, $\sigma_{h_{3}}$, $\sigma_{h_{4}}$	cm	0.25
	Probability (ϵ)	unitless	0.05

**Table 6:** MF-BSSTC design specifications for the simulation under measurement noise.

Subcontroller	Parameter	Value (Dimensionless)
$h_{3}$	$c_{1}$, $c_{2}$, $c_{3}$, |Δ|, $\lambda_{1}$, $\lambda_{2}$	1.20, 1.02, 1.00, 1.00, 1.50, 1.10
$h_{4}$	$c_{1}$, $c_{2}$, $c_{3}$, |Δ|, $\lambda_{1}$, $\lambda_{2}$	1.20, 1.05, 1.00, 1.00, 1.50, 1.10

**Table 7:** Performance indices.

Controller	Control Input	ITAE	IAE	ISE
MF-BSSTC	$u_{1}$	4711.8	77.1	659.0
	$u_{2}$	4176.1	101.4	786.7
PI	$u_{1}$	33,243.0	911.9	4564.3
	$u_{2}$	5549.4	387.2	1935.1
MPC	$u_{1}$	9997.1	250.9	1414.0
	$u_{2}$	2804.9	243.6	1176.9

## Data Availability

The data presented in this study are available on request from the corresponding author.
